# Supplementary material for: Identification of photoperiod-induced specific miRNAs in the adrenal glands of Sunite sheep (Ovis aries)
Source: Front Vet Sci. 2022 Jul 22;9:888207. doi: 10.3389/fvets.2022.888207 (PMC9354845; doi:10.3389/fvets.2022.888207)
Supplement: Supplementary file 1 [file Data_Sheet_1.ZIP › Supplementary file/miRDeep2 file.zip!/miRDeep2 file/Know_Structure/LP42A1/oar-mir-3957.pdf]

miRBase precursor : oar-mir-3957
Total read count : 55
oar-miR-3957-5p read count : 39
oar-miR-3957-3p read count : 15
remaining reads : 1

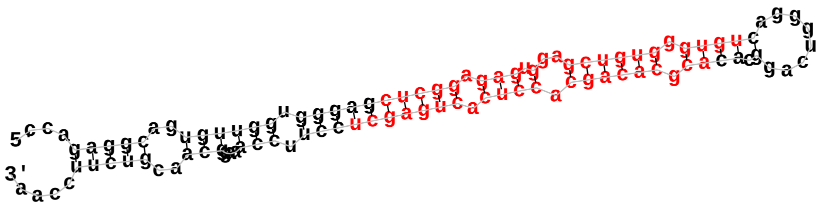

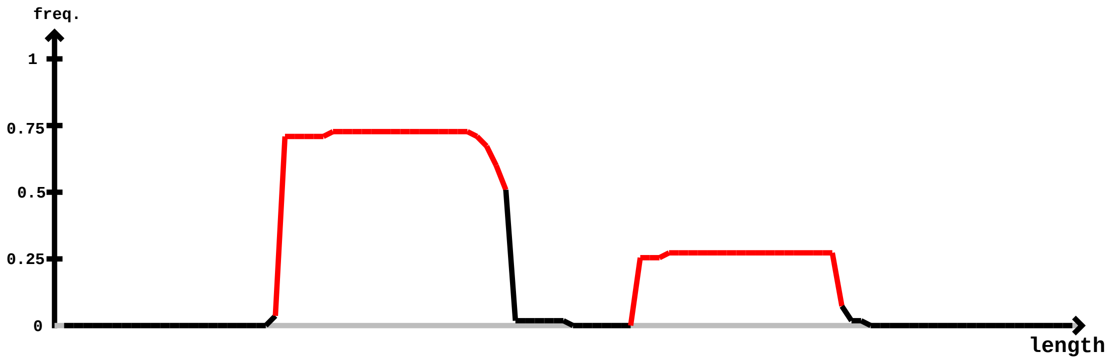


```
                                        oar-miR-3957-5p
                                                                      oar-miR-3957-3p
5'- ccagaggcaguguuggugggagcucggagaguggagcugugggugucagggucaggcacacgcacagcaccucacugagcuccuuccaaaggcaacgucuuccaa   -3'    exp
    ...(((((..((((((.((((((((((.(((.(..((((((.(((((........).)))).)))))).)))).)))))))))).)))...)))..)))))....   reads      mm         sample
    ....................gcucggagaguggagcugugg................................................................   1          0          seq
    ....................gcucggagaguggagcuguggg...............................................................   1          0          seq
    .....................cucggagaguggagcuguggg...............................................................   1          0          seq
    .....................cucggagaguggagcugugggu..............................................................   4          0          seq
    .....................cucggagaguggagcugugggug.............................................................   5          0          seq
    .....................cucggagaguggagcugugggugu............................................................   27         0          seq
    ..........................agaguggagcugugggugucagggu......................................................   1          0          seq
    ...........................................................acgcacagcaccucacugagc.........................   11         0          seq
    ...........................................................acgcacagcaccucacugagcu........................   3          0          seq
    ..............................................................cacagcaccucacugagcucc......................   1          0          seq
```
